# Validity and reliability of the arabic version of the HLS-EU-Q16 and HLS-EU-Q6 questionnaires

**DOI:** 10.1186/s12889-023-15226-5

**Published:** 2023-02-10

**Authors:** Lina Bergman, Ulrica Nilsson, Karuna Dahlberg, Maria Jaensson, Josefin Wångdahl

**Affiliations:** 1 Department of Neurobiology, Care Sciences and Society, Karolinska Institute, Stockholm, Sweden; 2 Department of Perioperative Medicine and Intensive Care, Karolinska University Hospital, Stockholm, Sweden; 3 Faculty of Medicine and Health, School of Health Sciences, Örebro University, Örebro, Sweden; 4 Aging Research Center, Karolinska Institute and Stockholm University, Stockholm, Sweden; 5 Department of Public Health and Caring Sciences, Uppsala University, Uppsala, Sweden; 6 Aging Research Center, Department of Neurobiology, Care Sciences and Society, Karolinska Institutet & Stockholm University, Tomtebodavägen 18a, 171 77 Solna, Sweden

**Keywords:** HLS-EU-Q, Comprehensive health literacy, General health literacy, Validation, Reliability, Arabic, Sweden

## Abstract

**Background:**

Health literacy is an important social determinant of health and affects the ability to make decisions and take action to manage one’s health. The purpose of this study was to psychometrically examine the Arabic versions of HLS-EU-Q16 and HLS-EU-Q6 and their response patterns among Arabic-speaking persons in Sweden.

**Methods:**

By convenience sampling from a variety of settings, a total of 335 participants were invited to participate. The participants completed a self-assessment of comprehensive health literacy by answering the Ar-HLS-EU-Q16 questionnaire, also including the six items for Ar-HLS-EU-Q6. Statistical analysis was guided by The COnsensus-based Standards for the selection of health Measurement Instruments. Floor/ceiling effects, construct, structural and criterion validity, test-retest reliability and internal consistency reliability were analysed.

**Results:**

In total, 320 participants were included in the psychometric evaluation. Mean age was 42.1 (SD 12.5), 63% (n = 199) were females and 53% (n = 169) had at least 10 years of education. No floor or ceiling effect were found for the Ar-HLS-EU-Q16 or Ar-HLS-EU-Q6. For both instruments, construct validity was confirmed in four out of five expected correlations (weak positive correlation to educational level, self-perceived health, and years in Sweden; moderate positive correlation with higher sum score on the Arabic electronic health literacy scale, and strong positive correlation to higher Ar-HLS-EU-Q16/Ar-HLS-EU-Q6). For Ar-HLS-EU-Q16, the principal component analysis resulted in a three-factor model with all items significantly correlating to only one factor. For Ar-HLS-EU-Q6, the principal component analysis supported a one-factor solution. Criterion validity showed poor agreement between the two questionnaires with a Cohen κ 0.58 (p < 0.001). Test-retest reliability showed a substantial agreement, Cohen’s κ for Ar-HLS-EU-Q16 and Ar-HLS-EU-Q6 were both 0.89. The internal consistency of both versions was acceptable, Cronbach alpha for Arabic-HLS-EU-Q16 was 0.91 and for Arabic-HLS-EU-Q6, 0.79. Split-half reliability was 0.95 and 0.78, respectively.

**Conclusion:**

The Arabic version of HLS-EU-Q16 shows good psychometric properties, validated in a Swedish setting. The findings can further inform and guide future validation studies in other settings worldwide. Furthermore, the results of the present study did not support criterion validity of Ar-HLS-EU-Q6.

## Background

Health literacy is an important social determinant of health [1] that more and more researchers and practitioners in various fields have become aware of and interested in studying and working with. Furthermore, at the societal level, many international health policies and guidelines emphasize the importance of promoting and taking into account health literacy in health promotion, disease prevention, and in the healthcare, to increase the equity in health and to reach the sustainable development goals in Agenda 2030 [2, 3].

However, the fact that there are many different types and dimensions of health literacy makes it important to clearly identify the definition used and to carefully consider the choice of instrument so that the health literacy that one is interested in is the one being measured. According to Sorensen et al. (2012) *“health literacy is linked to literacy and entails people’s knowledge, motivation and competences to access, understand, appraise, and apply health information in order to make judgments and take decisions in everyday life concerning healthcare, disease prevention and health promotion to maintain or improve quality of life during the life course”* [4]. This definition describes comprehensive health literacy (CHL), also called “general health literacy”, and is the definition of health literacy used in this study.

One of many instruments measuring CHL, which is frequently used worldwide, is the European health literacy survey questionnaire (HLS-EU-Q) [5, 6]. The original version of HLS-EU-Q consists of 47 items on which the respondents subjectively assess how difficult they perceive the statement corresponding to the item to be. The statements have to do with the respondent’s own perceived ability to obtain, understand, evaluate, and use health information regarding health promotion, disease prevention and health care, to maintain and promote health and are answered on a 4-point Likert scale. A fifth response option, ‘don´t know’ can also be used if data is collected orally, but is only used by the interviewer if respondents don´t answer [5]. However, an instrument with 47 items is rather cumbersome for the respondent to answer and therefore, the developers have constructed several shorter versions of this instrument, e.g. the HLS-EU-Q16 and the HLS-EU-Q6 (which consists of 16 respectively six items from the original HLS-EU-Q) [7]. The HLS-EU-Q16 has been translated into several languages and psychometrics tests show that the validity of most of the translated versions are satisfactory [8–14], only the Japanese version has shown weak validity [15].

Arabic is the sixth most common language worldwide [16] and the language that most migrants with a refugee background speak [17]. In 2014, the English version of HLS-EU-Q16 was adapted and translated into Arabic in line with with guidelines for the translation of questionnaires [18]. The original English version was independently translated into Arabic by two qualified translators. Two other Arabic-English-Swedish speaking people reviewed the translation individually and made suggestions for improvements to make the questions easier to understand. Together with the last author (JW), they then revised the translation and finalized the final Arabic version before it was retranslated into English by a second qualified translator. The instrument was then pilot tested through four cognitive interviews in which the Arabic-speaking participants spoke aloud about what they were thinking as they read and completed the questionnaire [19]. In the first translated Arabic version, the “don´t know” option was given as a fifth answer option, as well. However, since many choose this alternative, which is treated as a missing value in the analysis, an extensive proportion of the study population did not get a valid HLS-EU-Q16 index score, and thus had to be excluded from further analysis. Therefore, the “don’t know” option was removed, and instead, a four point Likert scale is used [7]. This version has subsequently been used in several studies, both in Sweden and internationally [20–24]. Thus, evaluating the validity and reliability of the Arabic HLS-EU-Q16 (Ar-HLS-EU-Q16) as well as the six-item version, the HLS-EU-Q6 (Ar-HLS-EU-Q6) is crucial when data is collected in written form.

## Methods

### Aim

The purpose of this study was to psychometrically examine the Arabic versions of HLS-EU-Q16 and HLS-EU-Q6 and their response patterns among Arabic-speaking persons in Sweden.

### Study design

The study had a prospective psychometric design and is a part of a larger project aiming to evaluate and subsequently measure CHL and electronic health literacy among Swedish and Arabic-speaking persons in Sweden [25–28]. The project has been approved by the Regional Ethical Review Board in Stockholm, Sweden (No. 2019/5:1). All respondents were informed in verbal and written form, in Arabic and Swedish, about the purpose and the procedures of the study, and told that it was voluntary to participate in the study, and that they could withdraw from it at any time. They were guaranteed confidentiality and secure data storage, and told that by answering the questionnaire, they were giving their informed consent to participate in the study.

### Sample, setting and data collection

The data collection was carried out from May to September 2019. Inclusion criteria were: 18 years of age or older, having Arabic as their mother tongue, and being present on the day of data collection. Convenience sampling was used, and the respondents were recruited by the last author visiting various arenas in three large Swedish cities, such as courses in civic orientation for newly-arrived refugees, an Arabic language school and some informal Arabic-language networks. For more details please, see Wångdahl et al. 2021 [26]. Information about the study was given on site, orally and through an information letter given on the same day as data collection at each arena.

The sample size of around 300 respondents was selected based on guidelines for psychometric testing of instruments [29]. According to this, 335 people were invited to participate in the study. Of those who were invited to participate in the study, 49 were asked to respond to the questionnaire twice at approximately 7-day intervals in order to examine the test-retest reliability. A sample size of at least 25 people has been suggested as applicable for evaluating test-retest reliability [30]. The oversampling was used in order to minimize the risk of having a too small test-retest group in the case of dropouts at the second assessment. To be able to combine the test-retest questionnaires, the respondents had to mark a study-specific personal code consisting of the first three letters of their mother’s name and the year he or she was born. Twelve participants were excluded from the analysis for different reasons, such as incomplete questionnaires or absence at the second measurement. The final test-retest group therefore came to consist of 37 respondents.

### Questionnaires

The Ar-HLS-EU-Q16 consists of 16 items. Each of the items have the following four response alternatives: very difficult –difficult –easy – very easy. In the analysis an HLS-EU-Q16 index score ranging from 0 to 16 is calculated (requires response on at least 14 items). This is done by first dichotomizing the response alternatives into difficult (difficult and very difficult) and easy (easy and very easy), giving difficult the value 0 and easy the value 1, and then adding up the values of all items. Thereafter, the study population was divided into sub-groups based on CHL level. Based on the recommendations of the developer, [31] the threshold values were set to: 0–8 for inadequate CHL, 9–12 for problematic CHL, and 13–16 for sufficient CHL.

The Ar-HLS-EU-Q6 consists of 6 items (included in Ar-HLS-EU-Q16) and the index score is calculated differently compared to the HLS-EU-Q16. First, each response alternative is coded separately (very difficult = 1, difficult = 2, easy = 3 and very easy = 4), then the values of each item are added together and divided by the total number of items (requires a response on at least 5 items). This gives the HLS-EU-Q6 index score. Based on the recommendations of by the developer [32] and previous research [10] the threshold values were set to: ≤2 for inadequate CHL, > 2 and ≤ 3 for problematic CHL, and > 3 for sufficient CHL.

The questionnaire also included demographic questions about age, biological sex, education level, country of birth, years lived in Sweden and health status. Health status was measured with the well-used and established question “How do you assess your overall health status? “ with its response options, “very poor, poor, fair, good, or very good” [33]. Electronic health literacy, i.e. “the ability to seek, find, understand, and appraise health information from electronic sources and apply the knowledge gained to addressing or solving a health problem” [34], was measured using the Arabic Electronic Health Literacy Scale (Ar-eHEALS) consisting of 8 items [26]. The items are answered on a Likert Scale ranging from 5 (strongly agree) to 1 (strongly disagree). The value of the items are added together to produce an Ar-eHEALS sum score [26].

### Psychometric testing and data analysis

The COnsensus-based Standards for the selection of health Measurement INstruments (COSMIN) guided the choice of correct psychometric tests [35–38]. Data are presented with both number and percentages or with mean, standard deviation (SD) and intervals, depending on what is appropriate based on the type of data. Potential differences in biological sex, age, years in Sweden, educational level and self-perceived health between participants with a valid, respectively non-valid, HLS-EU-Q16 index score, and between participants in the test and re-test group, was assessed using the chi-square test, independent sample t-test and Mann-Whitney U test. Two tailed p values < 0.05 was considered as statistically significant. Floor and ceiling effects (the number of respondents with the lowest or highest possible score on the instrument when answering), were examined by calculating the percentage of respondents who had those scores. If > 15% respondents have the lowest score, floor effect could be considered, and if > 15% respondents have the highest score, ceiling effect could be considered [38]. Frequency of missing data for each item was calculated and evaluated based on the criterion of < 5% [39].

Construct validity, which describes how well the results from an instrument are consistent with a hypothesis (i.e., assessing the concept that it is designed to measure) [36], was examined by analysing the associations between Ar-HLS-EU-Q16 index score, Ar-HLS-EU-Q6 index score, age, level of education, self-perceived health and electronic health literacy, by calculating Spearman’s rank correlation. Negative correlation between health literacy and high age [40–42], and positive correlations between health literacy and high level of education [22, 40, 41, 43], high self-perceived health [21, 40, 44], years in Sweden [27] and high electronic health literacy [25] have been found in previous studies. A correlation coefficient magnitude between 0 and 0.1 was viewed as negligible, between 0.1 and 0.39, as weak, between 0.4 and 0.69, as moderate, between 0.7 and 0.89, as strong, and between 0.9 and 1.0, as very strong [45]. Structural validity of Ar-HLS-EU-Q6 and Ar-HLS-EU-Q16 was assessed by a principal component analysis (PCA) as recommended in other validation studies [8, 14]. Sample adequacy and appropriateness of the data set was assessed using the Kaiser-Meyer-Olkin (KMO) index (criteria > 0.8) and Bartlett’s test of sphericity. Factor extraction was informed by the latent root criterion (eigenvalue > 1) and visual examination of the scree plot. As correlations between factors were expected, oblique (oblimin) rotation was used [46].

Criterion validity was examined by assessing the agreement between CHL levels defined by the Ar-HLS-EU-Q16 and CHL levels defined by Ar-HLS-EU-Q6 using the Cohen κ coefficient. The Cohen κ coefficient was also used to assess test-retest reliability, i.e., the agreement between the two points in time. A Cohen K coefficient value > 0.7 was considered acceptable [37]. Internal consistency reliability, (the correlation between the items in the instruments) were assessed using Cronbach α (> 0.7 indicating good reliability) [37]. Split-half reliability was calculated using Spearman’s-Browns coefficient with a reliability coefficient of 0.70 to 0.95 considered acceptable [37].

## Results

### Demographics of the sample

A total of 333 respondents participated in the study, of these, 4% (n = 13) were excluded due to more than two missing answers on item level for Ar-HLS-EU-Q16 (i.e. no valid Ar-HLS-EU-Q16 index score could be calculated). There was no significant difference between those with a valid HLS-EU-Q16 index score and those excluded due to age (p = 0.87), biological sex (p = 0.97), educational level (p = 0.33), years in Sweden (p = 0.88) or self-perceived health (p = 0.85).

The mean age of the 320 respondents included was 42.1 years (SD 12.5) and most were females (n = 199; 63%). Most of the respondents were born in Syria (n = 189; 59%) followed by respondents born in Iraq (n = 71; 22%). The number of years the respondents lived in Sweden varied between 0 and 38 years (SD 9.6). Around half of the respondents had graduated from university (n = 169; 53%) and two thirds of respondents perceived their own general health as good or very good (n = 214; 67%). The Ar-HLS-EU-Q16 mean index score was 11.2 (SD 3.7), and the Ar-HLS-EU-Q6 mean index score was 2.7 (SD 0.6). A higher proportion of respondents had a sufficient HL level (n = 124; 39%) according to Ar-HLS-EU-Q16 in comparison with respondents who had a sufficient HL according to Ar-HLS-EU-Q6 (n = 63; 20%). The respondents’ mean Ar-eHEALS sum score was 28.2 (SD 6.1). (Table 1).

**Table 1:** Demographics of the total sample (n = 320) and of the test-retest group (n = 37)

Characteristics	Total sample^a^	Test-retest group
Biological sex, n (%)		
Male	116 (37)	9 (24)
Female	199 (63)	28 (76)
**Age in years**		
Mean (SD)	42.1 (12.5)	46.7 (10.1)
Range	21–77	28–68
**Country of birth, n (%)**		
Syria	189 (59)	16 (43)
Iraq	71 (22)	12 (32)
Other	60 (19)	9 (25)
**Years in Sweden**		
Mean (SD)	9.6 (8.4)	11.6 (11.2)
Range	0–38	1–32
**Highest education level, n (%)**		
None	5 (2)	1 (3)
1–6 years	24 (8)	3 (8)
7–9 years	47 (15)	5 (14)
10–12 years	73 (23)	8 (22)
Graduated from university	169 (53)	20 (54)
**General self-perceived health, n (%)**		
Very poor	6 (2)	1 (3)
Poor	19 (6)	0 (0)
Fair	80 (25)	14 (38)
Good	135 (42)	14 (38)
Very good	79 (25)	8 (22)
**Ar-HLS-EU-Q16**^**b**^, **n (%)**		
Inadequate	66 (21)	4 (11)
Problematic	130 (41)	11 (30)
Sufficient	124 (39)	22 (59)
Mean index score (SD)	11.2 (3.7)	12.8 (3.4)
Range	0–16	2–16
**Ar-HLS-EU-Q6**^**c**^, **n (%)**		
Inadequate	38 (12)	5 (14)
Problematic	217 (68)	22 (59)
Sufficient	63 (20)	10 (27)
Mean index score (SD)	2.7 (0.6)	2.8 (0.6)
Range	1–4	1.8–4
**Ar-eHEALS** ^**d**^		
Mean sum score (SD)	28.2 (6.1)	27.8 (7.0)
Range	8–40	10–40

^a^Missing responses (biological sex, n = 5; age, n = 11; years in Sweden, n = 80; educational level, n = 2; general self-percivied health, n = 1; HLS-EU-Q6, n = 2; eHEALS, n = 31 in the total sample and years in Sweden, n = 11; eHEALS, n = 5 in the test-retest group were not included in the denominator when calculating percentages; ^b^The Arabic Health Literacy Survey European Questionnaire 16 items; ^c^The Arabic Health Literacy Survey European Questionnaire 6 items; ^d^The Arabic eHealth Literacy Scale

### Item distributional statistics and floor and ceiling effects

All items of Ar-HLS-EU-Q16 and Ar-HLS-EU-Q6 had full variance (i.e., at least one respondent for each scoring option) (Table 2). A ceiling effect was noted for 12 of 16 items with > 15% of the respondents scoring the highest possible score. One item, *Understand what your doctor says to you* had more than 5% missing (n = 24;8%). No other pattern of structural problems regarding difficulties in responding to certain items could be identified.

**Table 2:** Distribution of responses on items included in Ar-HLS-EU-Q16/Q6

Items, n (%)	Very difficult	Difficult	Easy	Very easy	Missing
a. Find information on treatments of illnesses that concern you	27 (8)	80 (25)	154 (48)	56 (18)	3 (1)
b. Find out where to get professional help when you are ill	37 (12)	125 (39)	119 (37)	37 (12)	2 (1)
c. Understand what your doctor says to you	15 (5)	76 (24)	140 (44)	65 (20)	24 (8)
d. Understand your doctor’s or pharmacist’s instruction on how to take a prescribed medicine	10 (3)	47 (15)	171 (53)	92 (29)	0 (0)
**e. Judge when you may need to get a second opinion from another doctor**	**46 (14)**	**141 (44)**	**105 (33)**	**26 (8)**	**2 (1)**
**f. Use information the doctor gives you to make decisions about your illness**	**18 (6)**	**101 (32)**	**154 (48)**	**42 (13)**	**5 (2)**
g. Follow instructions from your doctor or pharmacist	6 (2)	41 (13)	197 (62)	71 (22)	5 (2)
**h. Find information on how to manage mental health problems like stress or depression**	**40 (13)**	**109 (34)**	**130 (41)**	**31 (10)**	**10 (3)**
i. Understand health warnings about behaviour such as smoking, low physical activity and drinking too much	14 (4)	36 (11)	159 (50)	106 (33)	5 (2)
j. Understand why you need health screenings	11 (3)	44 (14)	171 (53)	90 (28)	4 (1)
**k. Judge if the information on health risks in the media is reliable**	**29 (9)**	**109 (34)**	**129 (40)**	**49 (15)**	**4 (1)**
l. Decide how you can protect yourself from illness based on information in the media	13 (4)	71 (22)	169 (53)	62 (19)	5 (2)
**m. Find out about activities that are good for your mental well-being**	**9 (3)**	**39 (12)**	**188 (59)**	**84 (26)**	**0 (0)**
n. Understand advice on health from family members or friends	8 (3)	34 (11)	178 (56)	99 (31)	1 (0)
**o. Understand information in the media on how to get healthier**	**15 (5)**	**67 (21)**	**171 (53)**	**65 (20)**	**2 (1)**
p. Judge which everyday behaviour is related to your health	12 (4)	47 (15)	186 (58)	74 (23)	1 (0)

Items included in Ar-HLS-EU-Q6 are highlighted in bold text.

On scale level, no floor or ceiling effect were found for the Ar-HLS-EU-Q16 or Ar-HLS-EU-Q6. For Ar-HLS-EU-Q16, four respondents (1%) had a minimum index score (i.e., 0) and 41 respondents (13%) had a maximum index score (i.e., 16). For Ar-HLS-EU-Q6, one respondent (0.3%) had a minimum index score (i.e., 1) and 13 respondents (4.1%) had a maximum index score (i.e., 4).

### Construct, structural and criterion validity

Ar-HLS-EU-Q16 showed a weakly positive correlation with higher education level, higher self-perceived health, years in Sweden, a moderately positive correlation to a higher Ar-eHEALS sum score, and a strongly positive correlation to a higher Ar-HLS-EU-Q6. Ar-HLS-EU-Q6 demonstrated a weakly positive correlation to a higher education level, higher self-perceived health, years in Sweden, a moderately positive correlation to a higher Ar-eHEALS sum score and a strongly positive correlation to higher Ar-HLS-EU-Q16. No correlation was found between Ar-HLS-EU-Q16/Q6 and higher age (Table 3).

**Table 3:** Spearman rho correlations between Ar-HLS-EU-Q16, Ar-HLS-EU-Q6, age, education, self-perceived health, Ar-eHEALS and years in Sweden

Variables	1.	2.	3.	4.	5.	6.	7.
1. Ar-HLS-EU-Q16^a^	1,000						
2. Ar-HLS-EU-Q6^b^	0.838^**^	1.000					
3. Age^c^	0.019	− 0.023	1.000				
4. Educational level^d^	0.262^**^	0.180^**^	− 0.007	1.000			
5.Self-percieved health^e^	0.344^**^	0.359^**^	0.321^**^	− 0.194^**^	1.000		
6. Ar-eHEALS sum score^f^	0.500^**^	0.492^**^	− 0.066	0.253^**^	− 0.299^**^	1.000	
7. Years in Sweden^g^	0.294^**^	0.292^**^	0.288^**^	0.223^**^	0.046	0.134^*^	1.000

^a^Ar-HLS-EU-Q16 index score, range 0–16; ^b^Ar-HLS-EU-Q6 index score, range 1–4; ^c^Age, range 21–77; ^d^Highest educational level, 1 = none, 2 = 1–6 years, 3 = 7–9 years, 4 = 10–12 years, 5 = Graduated from university; ^e^Self-perceived health, 1 = very poor, 2 = poor, 3 = fair, 4 = good, 5 = very good; ^f^Ar-eHEALS sum score, range 8–40; ^g^Years in Sweden, range 0–38.

* Correlation is significant at the 0.05 level (2–tailed) ** Correlation is significant at the 0.01 level (2–tailed). Abbreviations: Ar-HLS–EU-Q: Arabic Health literacy survey European questionnaire; Ar-eHEALS: Arabic Electronic Health Literacy Scale

In terms of structural validity of Ar-HLS-EU-Q16, the assumptions for PCA were met with a KMO index value of 0.907 and a significant Barlett’s test of sphericity (p < 0.001). A three-factor solution was supported by the latent root criterion (eigenvalues 6.9, 1.5 and 1) and visual examination of the scree plot. The three-factor model accounted for 59% of the variance. The items that clustered together suggest that factor 1 (item o, n, p, m, l, i, k, g and j) represents “Find, understand, and process information in connection to health”; factor 2 (item: e, b, f, a and h) represents “Find and process information in connection to health problems”; and factor 3 (item c and d) represents “Understand information from Healthcare Professionals” (Table 4). For Ar-HLS-EU-Q6, the PCA yielded a one-factor solution that explained 49% of the variance (KMO index value 0.798; Barlett’s test of sphericity p < 0.001) (Table 5).

**Table 4:** Factor loadings of Ar-HLS-EU-Q16 (PCA, Oblimin rotation, n = 320)

Item	Factor				Subscales
	1	2	3		
o. Understand information in the media on how to get healthier	0.791				Find, understand, and process information in connection to health
n. Understand advice on health from family members or friends	0.768				
p. Judge which everyday behaviour is related to your health	0.745				
m. Find out about activities that are good for your mental well-being	0.709				
L. Decide how you can protect yourself from illness based on information in the media	0.700				
i. Understand health warnings about behaviour such as smoking, low physical activity and drinking too much	0.682				
k. Judge if the information on health risks in the media is reliable	0.583				
g. Follow instructions from your doctor or pharmacist	0.499				
j. Understand why you need health screenings	0.475				
e. Judge when you may need to get a second opinion from another doctor		0.858			Find and process information in connection to health problems
b. Find out where to get professional help when you are ill		0.831			
f. Use information the doctor gives you to make decisions about your illness		0.684			
a. Find information on treatments of illnesses that concern you		0.543			
h. Find information on how to manage mental health problems like stress or depression		0.501			
c. Understand what your doctor says to you			-0.817		Understand information from Healthcare Professionals
d. Understand your doctor’s or pharmacist’s instruction on how to take a prescribed medicine			-0.808		

Abbreviations: Ar-HLS-EU-Q: Arabic Health literacy survey European questionnaire; PCA: Principal Component Analysis

**Table 5:** Factor loadings of Ar-HLS-EU-Q6 (PCA, n = 320)

Item	Factor
	1
h. Find information on how to manage mental health problems like stress or depression	0.742
m. Find out about activities that are good for your mental well-being	0.721
f. Use information the doctor gives you to make decisions about your illness	0.712
O. Understand information in the media on how to get healthier	0.707
k. Judge if the information on health risks in the media is reliable	0.666
e. Judge when you may need to get a second opinion from another doctor	0.665

Abbreviations: Ar-HLS-EU-Q: Arabic Health literacy survey European questionnaire; PCA: Principal Component Analysis

Criterion validity was assessed by examining the agreement between CHL levels defined by Ar-HLS-EU-Q16 respectively Ar-HLS-EU-Q6. This resulted in a Cohen κ of 0.58 (p < 0.001), in other words, a poor agreement between the two questionnaires.

### Test-retest reliability

The test-retest group consisted of 37 respondents. There was a significant difference in the distribution of age between the re-test group and the total sample, where the respondents in the retest group were older. There were no significant differences in the distribution of sex, years in Sweden, educational level and self-perceived health. Thus, the demographics of the two groups were rather similar (Table 1). Test-retest reliability showed a substantial agreement (Cohen’s κ > 0.7). The Cohen’s κ for Ar-HLS-EU-Q16 index score was 0.89, for Ar-HLS-EU-Q6 index score 0.89, for Ar-HLS-EU-Q16 levels 0.89 and for Ar-HLS-EU-Q6 levels 0.87.

### Internal consistency

The internal consistency of both versions was acceptable. Cronbach alpha for Ar-HLS-EU-Q16 index score was 0.91 and for Ar-HLS-EU-Q6 index score 0.79. Split-half reliability according to the Spearman-Browns coefficient was 0.95 for Ar-HLS-EU-Q16 and 0.78 for HLS-EU-Q6.

## Discussion

The aim of the present study was to examine the psychometric properties of the Arabic versions of HLS-EU-Q16 and HLS-EU-Q6 among Arabic speaking persons in Sweden. Our results indicate that the Ar-HLS-EU-Q16 has good psychometric properties, validated in a Swedish setting. However, the psychometric properties of the Ar-HLS-EU-Q6 were questionable as criterion validity could not be supported.

Our study findings showed that all items on Ar-HLS-EU-Q16/6 had full variance, but 12 out of 16 items showed ceiling effects. One item *Understand what the doctor says to you* had missing responses of more than 5%. Early development and validation work of the HLS-EU- Q [5, 7] support the applicability of this item in measuring CHL. Also, in examining the psychometric properties of HLS-EU-Q16/6 in a native Swedish speaking population, no missing responses were detected for the present item (Bergman et al. 2023 in manuscript). Thus, one possible explanation for the slightly higher proportion of missing responses to item could be the discomfort some members of our study population might feel when answering the question [39]. Another possible explanation could be problems interpretating the item. In Sweden, persons that do not speak or understand Swedish are, if needed, entitled to a professional translator during their contacts with healthcare service [47]. This means that this item could be interpreted differently. For example, does it mean understanding what the doctor says with an interpreter present or without one? This was also noted by the data collector since some participants asked for a clarification of this particular item.

In the present study, we identified significant relationships between CHL and education level, self-perceived health, electronic health literacy and years in Sweden. Thus, we were able to confirm four of our five expected correlations (i.e., 80%), which provides evidence of construct validity of Ar-HLS-EU-Q16/6 as > 75% of the correlations correspond with the hypothesises stated in advance [37]. In contrast to previous validation studies of HLS-EU-Q16 [9, 10], we identified a relationship between CHL and education level. Yet, our findings are in line with previous research showing an association with educational level and CHL [22, 40]. Notably, our hypothesis that CHL would be inversely related with age was not supported as no significant correlation could be detected. The relationship between age and health literacy has previously been identified in several studies [9, 40, 42]. For example, Sorensen et al. [40] identified older people as vulnerable for having limited health literacy in a study across eight European countries and Palumbo et al. [42] found a small negative correlation between age and health literacy skills in an Italian population. The discrepancy between present study findings and previous research might be due to the relatively young sample represented in our study (mean age 42.1, range 21–77). For example, compared to Palumbo et al’s study where 7% of the total number of the participants was 75 years or older.

In the present study, the PCA yielded a defined structure of Ar-HLS-EU-Q16 with all items loading significantly (> 0.4) to only one factor. The factor structure and factor loading pattern found in our sample of Arabic speaking persons in Sweden differs from the original model [5], as well as from patterns obtained in the Islandic [8] and the Romanian version [14] of HL-EU-Q16. These findings suggest that neither the domains nor the competencies of CHL that underlie the questionnaire manifest the same way across cultures [40]. Based on our findings, we do not recommend HLS-EU-Q16 to be divided into subscales and thus only a HL-EU-Q16 sum score should be calculated in assessing one’s CHL, which is in line with the recommendations from the developer [7]. For Ar-HLS-EU-Q6, the PCA supported a one-factor model with all items significantly loading in to one component. Our findings thus support the structural validity in terms of unidimensionality of HLS-EU-Q6. However,, few studies have yet evaluated the psychometric properties of the HLS-EU-Q6, and their results differ. The Italian version was assessed as reliable and valid [11] as was the Portuguese [48], but the French version failed to demonstrate unidimensionality, which led to the conclusion that its validity was questionable [10].

In the present study, we used HLS-EU-Q16 as our gold standard when assessing criterion validity. HLS-EU-Q16 is short form of HLS-EU-Q47, which to date does not exist in Arabic. However, items for HLS-EU-Q16 were selected using Rasch analysis in a large European sample, and its validity had been confirmed in further studies [49]. Correlations with the long form were high (r = 0.82) and HL levels between HLS-EU-Q47 and HLS-EU-Q16 corresponded in 76% of the cases [49]. In our study sample of Arabic speaking persons in Sweden, 124 (39%) had a sufficient CHL level accordingly to Ar-HLS-EU-Q16, but only 63 (20%) respondents were classified as having sufficient CHL accordingly to Ar-HLS-EU-Q6. Furthermore, the agreement between CHL levels detected by Ar-HLS-EU-Q16 and Ar-HLS-EU-Q6 was poor (Cohen κ = 0.58) and thus criterion validity of Ar-HLS-EU-Q6 could not be supported. Our results are in line with Roquette et al., which reported poor agreement (Cohen κ = 0.36) of CHL levels measured by HLS-EU-Q16 and HLS- EU-Q6 in an Arabic-speaking French population [10]. A validation study of HLS-EU-Q6 in an Italian population produced slightly better results, but still not satisfactory, with a concurrent classification between the two tests of 72.6% [11]. In contrast, Spearman correlations of HLS-EU-Q16 and HLS-EU-Q6 index scores were high, both in the present study and in the study by Rouquette et al. [10] (r = 0.84 and r = 0.88, respectively). This finding could be explained by the fact that correlation analysis does not consider the intervals between the variables and therefore Cohen’s K coefficient provides a better assessment of agreement [38]. Thus, the poor Kappa value indicates that thresholds should not be calculated for HLS-EU-Q6, or the results of such calculation should be carefully interpreted. In our study sample, four out of the six items included in Ar-HLS-EU-Q6 had the highest proportion of “very difficult” and “fairly difficult” answers (Table 2). One possible explanation why fewer persons are classified as having sufficient CHL when Ar-HLS-EU-Q6 is used could be that items included represents attributes of the trait (i.e., CHL) that are generally perceived as more difficult. Another possible explanation could be that threshold values are calculated differently between HLS-EU-Q16 and HLS-EU-Q6, with the former using dichotomized sum scores and the second using mean scores. [31, 32]

In terms of test-rest reliability, our study findings showed substantial agreement between the points in time for Ar-HLS-EU-Q16/6, both regarding index scores (0.89 and 0.89, respectively) and for CHL levels (0.89 and 0.87, respectively). Additional, internal consistency for both scales was acceptable with alpha values > 0.7. Thus, score differences in our study sample were low, indicating that the instruments are stable over time and that reproducibility is high [37, 39]. To our knowledge, this is the first study investigating test-retest reliability for HLS-EU-Q6. However, our results resonate with previous research indicating high reliability and stability for HLS-EU-Q16 [12]. The internal consistency for the Ar-HLS-EU-Q6/16 was acceptable (α = 0.91 and α = 0.79, respectively), which is in line with results from the Icelandic [8], Italian [11], French [10], and Japanese [15] versions of HLS-EU-16, and the French [10], Italian [11] and Portuguese/Brazilian [48] versions of HLS-EU-Q6.

In the present study, the Arabic versions of HLS-EU-Q16/6 were evaluated in a Swedish context. Importantly, although HLS-EU-Q16 has previously been validated among Arabic speaking persons [19] and used in research investigating CHL in Arabic populations [22, 44, 50], cross-cultural adaption and validation is important to ensure that the instrument is psychometrically sound in the context in which it is intended to be used. One potential limitation of the questionnaire is that the items can be interpreted differently. When used among refugees and migrants, one might refer to the healthcare settings in their original country, or assume the presence of a professional translator, when answering the questions. We therefore suggest future researcher and users in clinical practice to clearly state the purpose and setting prior to handling out the questionnaire. Also, in this study, data was collected using a paper and pen questionnaire. The instrument was originally intended to be used by face-to-face interviews [5].The later might be beneficial as the test person can clarify any potential interpretation issues for each item. On the other hand, the presence of a test interviewer is time consuming and costly. For that reason, it is important to establish feasible, valid, and reliable ways of self-evaluation of CHL to further enhance widespread implementation and use in clinical practice.

The following limitations should be acknowledged when interpreting the results. First, this study used a convenience sampling approach and participants were recruited from the three largest cities in Sweden. The sample might therefore not be representative of Arabic speaking persons living in rural parts of Sweden. However, recruitment was performed in several arenas aiming for a diversity in age, biological sex, length of stay in Sweden, and level of education. Second, we used self-administered paper and pen questionnaire, thus restricting participation to persons who can read and understand Arabic. Using face-to-face interviews might enhance the inclusion of persons with limited functional literacy. As discussed above, there is thus a need of feasible, reliable and valid ways to collect self-reported data that do not require the presence of a test interviewer and our findings support the psychometric properties of a self-administrated paper and pen version of Ar-HLS-EU-Q16. Third, this study did not further examine construct validity of Ar-HLS-EU-Q6 using confirmatory factor analysis or Rasch modelling. We therefore suggest future studies continue to examine the validity of Ar-HLS-EU-Q6 using complementary methodological approaches. Yet, we were able to assess stability over time for HLS-EU-Q16/6, which adds valuable information about the overall psychometric properties of the instruments.

## Conclusion

Our findings support the psychometric properties of the Ar-HLS-EU-Q16, and we can therefore recommend that it be used to evaluate CHL among Arabic speaking persons in Sweden. The findings can further inform and guide future validation studies in other settings worldwide. That said, however, the results of the present study did not support criterion validity of Ar-HLS-EU-Q6. A major concern is that the instrument shows limitations in distinguishing between CHL levels. Thus, there is a risk that a person’s CHL level is not correctly classified into inadequate, problematic, or sufficient. This presents a problem in using that instrument in assessing CHL, especially in clinical practice. However, our findings tend to indicate that the Ar-HLS-EU-Q6 is reliable and that it has stability over time. One of the benefits of using a six-item questionnaire is that it is less time consuming. We suggest that Ar-HLS-Q6 should be used for research purposes only if mean values are calculated and, when appropriate, compared either between groups of participants or over time. However, further studies are needed, to specifically establish validity and accuracy of thresholds of the HLS-EU-Q6 before widespread use in clinical practice.

.

## Data Availability

The datasets used and/or analysed during the current study are available from the corresponding author upon reasonable request.

## List of abbreviations

Ar-HLS-EU-Q: Arabic European Health Literacy Survey Questionnaire
Ar-eHEALS: Arabic Electronic Health Literacy Scale
CHL: Comprehensive health literacy
COSMIN: The COnsensus-based Standards for the selection of health Measurement Instruments
HLS-EU-Q: European Health Literacy Survey Questionnaire
KMO: Kaiser-Meyer-Olkin
PCA: Principal Component Analysis
